# Preliminary analysis of the transcriptome of salivary glands of *Ornithodoros brasiliensis* (Acari: Argasidae)

**DOI:** 10.1186/1753-6561-8-S4-P145

**Published:** 2014-10-01

**Authors:** DNS Giovanni, GA Landulfo, LL Duarte, FF Santos, PL Ho, RZ Mendonça, E Carvalho, ILM Junqueira-de-Azevedo, LAB Proença, DM Barros-Battesti

**Affiliations:** 1 Laboratório de Parasitologia, Instituto Butantan, São Paulo, Brazil; 2 Laboratório Especial de Coleções Zoológicas, Instituto Butantan, São Paulo, Brazil; 3 Laboratório de Biotecnologia, Instituto Butantan, São Paulo, Brazil; 4 Laboratório Especial de Toxinologia Aplicada, Instituto Butantan, São Paulo, Brazil

## 

*Ornithodoros brasiliensis *Aragão is an endemic tick to Brazil, restricted to highlands of the state of Rio Grande do Sul[1]. It is a very aggressive species to humans, causing fever, great pain and intense inflammatory response at the bite site[2]. To build a cDNA library from the salivary glands of this tick. Pirosequencing was used, Model GS-454 Junior to sequencing the cDNA obtained from mRNA from the salivary glands, producing a total of 81,178 sequences (reads) with an average size of 400.8 bases. Were assembled 4,558 contigs using the program Genomics CLC-(CLCBio) and annotated by software Blast2GO, based on sequence similarity. These were compared to various banks (GenBank, GO, Enzyme Codes, InterPro, KEGG). The results showed that 20.93% of them were classified as belonging to the secretory pathway and 14.77% as proteins of membrane, confirming the tissue of salivary gland. The main families of transcribed genes were lipocalins (15.6%), moubatins which can be sub-classes of lipocalins (6.23%), protein with a tail acid (1.63%), and metalloproteases (1.23%). The sequences of interest present in the salivary glands were cloned into bacterial heterologous system[3-5]. The recombinant proteins were expressed in sufficient quantities to analyze the sequences of lipocalins, moubatins, metalloproteases, cell cycle modulating and related to analgesia. All these proteins show functions that modify the coagulation activities, modulating the immune response and inhibition of platelet aggregation, among others, showing their potential for development of bioproducts with medical and industrial interests.
